# Synergism between Hedgehog-GLI and EGFR Signaling in Hedgehog-Responsive Human Medulloblastoma Cells Induces Downregulation of Canonical Hedgehog-Target Genes and Stabilized Expression of GLI1

**DOI:** 10.1371/journal.pone.0065403

**Published:** 2013-06-10

**Authors:** Frank Götschel, Daniela Berg, Wolfgang Gruber, Christian Bender, Markus Eberl, Myriam Friedel, Johanna Sonntag, Elena Rüngeler, Hendrik Hache, Christoph Wierling, Wilfried Nietfeld, Hans Lehrach, Annemarie Frischauf, Reinhard Schwartz-Albiez, Fritz Aberger, Ulrike Korf

**Affiliations:** 1 Division of Molecular Genome Analysis, German Cancer Research Center (DKFZ), Heidelberg, Germany; 2 Department of Molecular Biology, University of Salzburg, Salzburg, Austria; 3 Division of Translational Immunology, German Cancer Research Center (DKFZ), Heidelberg, Germany; 4 Department of Vertebrate Genomics, Max Planck Institute for Molecular Genetics, Berlin, Germany; University of South Florida College of Medicine, United States of America

## Abstract

Aberrant activation of Hedgehog (HH) signaling has been identified as a key etiologic factor in many human malignancies. Signal strength, target gene specificity, and oncogenic activity of HH signaling depend profoundly on interactions with other pathways, such as epidermal growth factor receptor-mediated signaling, which has been shown to cooperate with HH/GLI in basal cell carcinoma and pancreatic cancer. Our experimental data demonstrated that the Daoy human medulloblastoma cell line possesses a fully inducible endogenous HH pathway. Treatment of Daoy cells with Sonic HH or Smoothened agonist induced expression of GLI1 protein and simultaneously prevented the processing of GLI3 to its repressor form. To study interactions between HH- and EGF-induced signaling in greater detail, time-resolved measurements were carried out and analyzed at the transcriptomic and proteomic levels. The Daoy cells responded to the HH/EGF co-treatment by downregulating GLI1, PTCH, and HHIP at the transcript level; this was also observed when Amphiregulin (AREG) was used instead of EGF. We identified a novel crosstalk mechanism whereby EGFR signaling silences proteins acting as negative regulators of HH signaling, as AKT- and ERK-signaling independent process. EGFR/HH signaling maintained high GLI1 protein levels which contrasted the GLI1 downregulation on the transcript level. Conversely, a high-level synergism was also observed, due to a strong and significant upregulation of numerous canonical EGF-targets with putative tumor-promoting properties such as MMP7, VEGFA, and IL-8. In conclusion, synergistic effects between EGFR and HH signaling can selectively induce a switch from a canonical HH/GLI profile to a modulated specific target gene profile. This suggests that there are more wide-spread, yet context-dependent interactions, between HH/GLI and growth factor receptor signaling in human malignancies.

## Introduction

During the last decade, it has become obvious that progression and severity of malignant diseases is often not caused by a single genetic aberration or deregulation of a single signaling pathway, but actually requires the cooperation of oncogenic-signaling pathways in cancer cells. For instance, Hedgehog (HH)/GLI and EGF-driven signaling can synergize and promote events, such as neural stem cell proliferation, as well as tumor initiation and progression [1] [2] [3] [4] [5] [6] [7]. Deregulation of at least one of the two pathways has been implicated in about one-third of all cancers, and frequently, both pathways are found aberrantly activated in the same tumor. This understanding has helped to develop the hypothesis that a simultaneous activation of both pathways can drive tumor development. Although HH- as well as EGF-mediated signaling have been intensely studied, the details of how signals derived from HH or EGF are integrated at the molecular level still needs to be clarified for distinct cell types, and in different cancer entities [7] [8] [9]. The first insights into HH/GLI and EGF crosstalk in cancer was provided by Kasper et al. and Schnidar, et al., who pointed out that co-activation of both pathways results in the induction of a specific gene expression pattern, which induces malignant transformation of human keratinocytes [4] [6]. The hypothesis that both pathways merge at the level of transcriptional regulation was also supported by other studies, which showed that several different genes indeed possess binding sites for GLI and EGF-regulated transcription factors, such as c-JUN/AP-1 [4] [6] [10]. Evidence for cooperative effects was also obtained at the level of protein activation, by demonstrating that GLI1 transcription factors need to be stabilized by MAPK and PI3K/AKT-signaling [11] [12], which also presented a prerequisite for cytoplasmic/nuclear shuttling of GLI proteins [13]. Finally, Whisenant et al. revealed a direct phosphorylation of GLI1 by ERK, which was anticipated as the explanation for the modified transcriptional activity of GLI proteins upon activation of MAPK and PI3K/AKT signaling [13] [5] [14]. The interaction of HH/GLI with EGF-induced signaling has been described in a number of tumor types such as skin, prostate, and pancreas [15] [16] [7] [17], while other kinases such as PKC [18] and mTOR/S6K [19] also positively regulate GLI activity.

The lack of human cancer cell lines clearly responsive to HH stimulation frequently complicates an unambiguous interpretation at the molecular level. The fact that many studies have been carried out in murine fibroblast cell lines, and have been based on overexpression of HH pathway components raises the question about the possible physiological relevance for the analysis of human cancer. Given this, we screened human cell lines for their SMO expression level and identified high level SMO expression in the Daoy human medulloblastoma (MB) cell line. This cell line revealed in further experiments a fully HH-responsive signaling pathway, which allowed for the interrogation of HH-associated signaling mechanisms under physiological conditions without the need to over-express HH-pathway proteins or use other artifact-prone perturbations. Employing a combination of high-throughput transcriptomics and validation of selected target genes on the protein level, we described the novel effects of HH-EGFR crosstalk on selective target gene expression. In contrast to human keratinocytes and pancreatic cancer cells, in medulloblastoma cells we also observed a repression of canonical HH-target genes while selected EGFR target genes were synergistically induced which can potentially contribute to the formation of a tumor-promoting microenvironment.

## Materials and Methods

### Cell Culture

Daoy cells (ATCC: HTB-186) and HEK293FT cells (ATCC: CRL-1573) were cultivated in Dulbecco’s modified Eagle’s medium (DMEM, Gibco/Invitrogen) with 10% (v/v) fetal bovine serum (FBS, Gibco/Invitrogen). Hyperconfluent Daoy cells were pre-starved for 24 h in serum-reduced DMEM medium, containing 0.5% (v/v) FBS, after which, we added Sonic Hedgehog-conditioned medium (Shh-N), EGF (5 ng/ml), AREG (5 ng/ml) or Smoothened Agonist SAG (100 nM) (Axxora LCC) for stimulation. To inhibit HH signaling, Cyclopamine (LC Laboratories) was added to the media at a final concentration of 5 µM. To inhibit PI3K/AKT signaling PI103 [1 µM] and LY294002 [20 µM] were used. MEK/ERK signaling was inhibited using U0126 [10 µM] and PD98059 [20 µM]. Cells were exposed to drugs 1 h prior to EGF stimulation.

### Preparation and Characterization of Shh-N Enriched Medium

Shh-N-conditioned medium was produced using HEK293FT cells (ATCC) by transient transfection with a Shh-encoding plasmid (kind gift of R. Wechsler-Reya) using Fugene® as previously described [20]. To monitor Shh-N synthesis and secretion, the conditioned medium was analyzed by Western blot, and detected with an anti-Shh antibody (2287, CST). The biological activity of the Shh-N enriched medium was assayed by adding Shh-N conditioned medium to SHH-Light II cells (ATCC: CRL-2795) [21] using a Luciferase assay. Briefly, 1*10^5^ SHH-Light II cells were cultivated in DMEM supplemented with 10% FBS, 0.4 mg/ml G418 and 0.15 mg/ml Zeocin and seeded in 12-well plates. SHH-Light II cells were treated for 48 hours with different concentrations of Shh-N conditioned medium, 5E1 Hedgehog blocking antibody [22], or combinations of both. SHH-Light II cell lysates were assayed for renilla and firefly luciferase activity, using a microplate reader (Tecan, Infinite® 200). Firefly values were normalized to renilla measurements, and reported as fold-changes (figure S1).

### RNA Isolation

Total RNA was obtained at 14 different time points 24 hours after EGF stimulation, and extracted using the RNeasy Mini kit (Qiagen, 74104), according to the manufacturer’s protocol. Quantity and purity of RNA was determined by measuring the optical density at 260 and 280 nm with a UV/Vis Spectrophotometer (Thermo Scientific, Nanodrop 1000) and BioAnalyzer 2100 (Agilent Technologies Deutschland GmbH).

### Illumina CHIP-based Gene Expression Analysis

500 ng of total RNA in 11 µl RNase free water served as starting material for the generation of biotin-labeled cRNA with the Illumina® TotalPrep™ RNA amplification kit, following supplier instructions. cRNA was cleaned up with cRNA filter cartridges before use for subsequent hybridization on Illumina® Sentrix BeadChips. To perform whole genome expression analysis HumanHT-12 v4, chips were incubated with biotin labeled cRNA for 18 h at 58°C in a hybridization oven under humidity-controlled conditions. After hybridization, the Illumina® Sentrix BeadChips were washed using buffers provided by the kit. 2.5 µl (1 mg/ml) of Streptavidin-Cy3 (per Chip) diluted in 2.5 ml Blocking buffer were incubated on a Chip for 10 minutes under gentle shaking conditions to allow binding of cRNA to gene-specific probes. After washing, Illumina® Sentrix BeadChips were dried and scanned. After performing image data analysis using Illumina’s BeadStudio to quantify gene expression signal levels, we applied quantile normalization across samples using the ‘lumi’ package in Bioconductor. Normalized signal intensities from each independent biological experiments were used to calculate fold-change ratios, and were compared with a control sample as reference. Data was submitted to GEO (accession number GSE46045).

### Identification of Cooperation Response Genes (CRG) and Calculation of Synergy Scores

For each gene, a linear regression model was set up with its gene expression “y” as response variable, and measurement time points and treatments as experimental variables. Model fitting was done in the statistical computing environment R [23], according to the following model “y ∼ time+treatment”. Four treatment groups were set up: control, Shh-N(GLI), EGF and Shh-N(GLI)+EGF. To identify significantly differing profiles of pairs of treatments (e.g. control vs. EGF), linear model fits were computed for the treatment pairs of interest. F-tests were used to test whether the additional amount of variance explained by the treatment variable (in addition to the time control variable) was significant at level *α = 0.05*. P-values were adjusted using the Benjamini and Hochberg method to correct for multiple testing [24]. All probes with a significant treatment effect (*p<α*) were stored as “affected probes” for the following comparisons: (1) EGF vs control (8,715 probes); (2) Shh-N vs control (2,111 probes); (3) EGF+Shh-N vs control (11,073 probes); (4) EGF+Shh-N vs EGF (8,663 probes), (5) EGF+Shh-N vs Shh-N (8,911 probes). To identify synergistic effects of the co-treatment Shh-N(GLI)+EGF in comparison to single treatments, probes that were differentially regulated according to comparisons (1) to (3), (4), and (5) were chosen as follows: probes identified in (1) to (3) were unified, and the intersection with probes identified by combining (4) and (5) was determined. The candidate probe set comprised the intersection of both groups (4,580 transcripts, 3,827 genes). Finally, a published algorithm was used as a basis [25] to rank synergistically regulated probes, and fold-changes were calculated for each time point. Synergy scores were defined as *(a+b)/d* with *a* as the expression level after EGF stimulation, *b* as the expression values obtained for Shh-N stimulation, and *d* as the condition of the co-stimulation. No synergism was concluded if the resulting reference value was <1, and synergistic effects were concluded for scores > = 1. To obtain the final synergy score in case data for several Illumina probes matching a certain target gene were available, the minimum of all scores calculated for each time point was chosen.

### cDNA Synthesis and Taqman Real Time PCR

Single strand cDNA synthesis was carried out using the RevertAid™ First Strand cDNA Synthesis kit (Fermentas, K1621). 1 µg of total RNA was mixed with 1 µl oligo (dT)_18_ primer and RNAse free water to a final volume of 11 µl. This mixture was incubated for 5 min at 70°C. Subsequently, a mastermix containing 4 µl of 5× reaction buffer, 2 µl of 10 mM dNTP, 1 µl of Prime RNase-Inhibitor (30 U/µl) and 1 µl of RevertAid RTase (200 U/µl) was added. Thereafter, all samples were kept at 37°C for 5 min, followed by incubation at 42°C for 1 h. The reaction was terminated by heating samples for 10 min at 70°C. cDNA was stored at −20°C. An equivalent of 25 ng RNA of each sample was used for Taqman Real Time PCR in combination with the UPL-Probe® system. The estimated expression value of analyzed target genes was normalized according to the housekeeping gene HPRT. Finally, the normalized expression values of each time point/treatment were used to calculate fold-change ratios, and compared to a control treatment sample which served as a reference. All experiments were performed, using cDNA from three independent biological experiments with technical duplicate samples (Taqman Primers including information on sequences and appropriate UPL-Probes are listed in Table S1.).

### Western Blot

Western blots were carried out, using 20 µl total protein lysate obtained from Daoy cell Laemmli or MPER lysates (40 µl buffer per 6-well). Primary antibodies (diluted 1∶1,000) directed against phospho-EGFR(Tyr 1173) (4407), phospho-AKT(Ser473) (4058), phospho-ERK1/2(Thr202/Tyr204) (4370), phospho-S6 (Ser235/236) (4858), phospho-cJUN(Ser73) (9164), and GLI1 (3538), were purchased from Cell Signaling Technologies (CST), anti-GLI2 antibody (sc-28674) from Santa Cruz Biotechnology (Santa Cruz, CA, USA). Western blots were carried out as previously described [26]. The monoclonal antibodies directed against GLI3 (6F5) was kindly provided by Dr. Suzie Scales of Genentech, Inc., San Francisco, CA, USA [27].

### Reverse Phase Protein Microarrays (RPPA)

Cell lysates were prepared using the Pierce NE-PER kit. The cytoplasmic fraction was analyzed by RPPA. Total protein concentration was determined using the BCA Protein Assay Kit (Pierce; 23225), and adjusted to a total protein concentration of 2 µg/µl. Prior to printing, Tween-20 was added to samples to create a final concentration of 0.05 (v/v) %. All samples were printed with two depositions as technical triplicates onto nitrocellulose-coated glass slides (Oncyte® Avid, Grace-Biolabs, #305278), using a contact spotter (2470 Arrayer, Aushon Biosystems). Blocking, target protein detection, scanning and data analysis was carried out as described beforehand [28] [26].

### Fluorescent ELISA (FLISA)

Quantitative protein concentrations were determined in ng/ml for EGF (MAB636, BAF236), MMP7 (MAB9072, BAF907), and IL-8 (MAB208, BAF208), using ELISA antibody pairs from R&D Systems. In brief, micro assay 96-well plates (Greiner Bio-One, #655097) were coated with 2 µg/ml of MAB9072 or 4 µg/ml of MAB636 or MAB208, and incubated o/n. Wells were blocked using 1% (w/v) BSA (ultrapure, Ambion, #AM2616) in PBS for 2 h. 100 µl of standard or sample (medium supernatant) was added in the capture step, and incubated for 2 h. 0.1 µg/ml BAF236 or 0.2 µg/ml BAF907 and BAF208 were used for analyte detection. Signal visualization was carried out by incubating with Alexa Fluor® 680 streptavidine (Molecular Probes, S-32358) for 1 h. All incubation steps were carried out at RT with adequate washing, using PBS +0.05% (v/v) Tween. After the last washing step, the wells were emptied and dried out by bottom-up centrifugation (2 min at 1,200 rpm). Finally, fluorescence signal intensity was determined at 700 nm using the Odyssey Scan System (LI-COR) with 84 µm resolution, plate offset of 4.0 and scan intensity set to 9.

### Preparation of Cell Supernatant used in the Tube Formation Assay

Daoy cells were treated with EGF alone, Shh-N alone, in a combination employing both ligands, and in a control without ligands. Supernatants were collected 24 hours after the initiation of EGF signaling. Supernatants were then concentrated 10-fold using Amicon ® Falcons (Amicon® Ultra-4; Millipore #UFC800596). Using Fluorescent ELISAs (FLISA), VEGFalpha, IL8, EGF and AREG, amounts were determined in the concentrated supernatants.

### Tube Formation Assay

Normal Human Dermal Fibroblasts (NHDF) (Promocell, Germany, Heidelberg) were seeded in 24-well plates, and cultured for 5 days in DMEM supplemented with 10% fetal calf serum, 1% HEPES and 1% non-essential amino acids. Human umbilical cord vein endothelial cells (HUVEC) (Promocell, Germany, Heidelberg) were seeded on the confluent NHDF layer, and either VEGF (1.25, 5, 10, 20 ng/mL), controls or cell culture supernatants in concentrations as indicated were added to the cell culture. Growth medium, including growth factors and test substances, was replaced at Day 4 of cell culture. At Day 8, medium was removed, cells were washed, and fixed in 70% EtOH (v/v) for 30 min at RT, followed by a washing step and incubation in MeOH/30% H_2_O_2,_ 40∶1 (v/v) for 10 min at RT. The cells were washed, incubated with a monoclonal antibody against the CD31 antigen (Dako, Hamburg, Germany), which is specifically expressed on endothelial cells, diluted 1∶40 for 30 min, then followed by a washing step incubated with a secondary goat anti-mouse IgG antibody coupled to biotin (Dako) for 20 min followed by a washing step and incubation with streptavidin coupled to horseradish peroxidase for 20 min. Antibody reactivity was visualized by adding AEC (3-amino-9-ethylcarbazole) chromogen substrate (Dako) to the cells for 14 min in the dark. The enzymatic reaction was then stopped by washing with water. The wells were sealed with mounting medium and microscopic quantitative analysis of tube formation was performed with the Angiosys 1.0, TCS (Cellworks) software.

## Results

### Daoy Cells Respond to Shh-N and to Smoothened Agonist with GLI3 Processing and Upregulation of Canonical HH/GLI Target Genes

**Figure 1:**
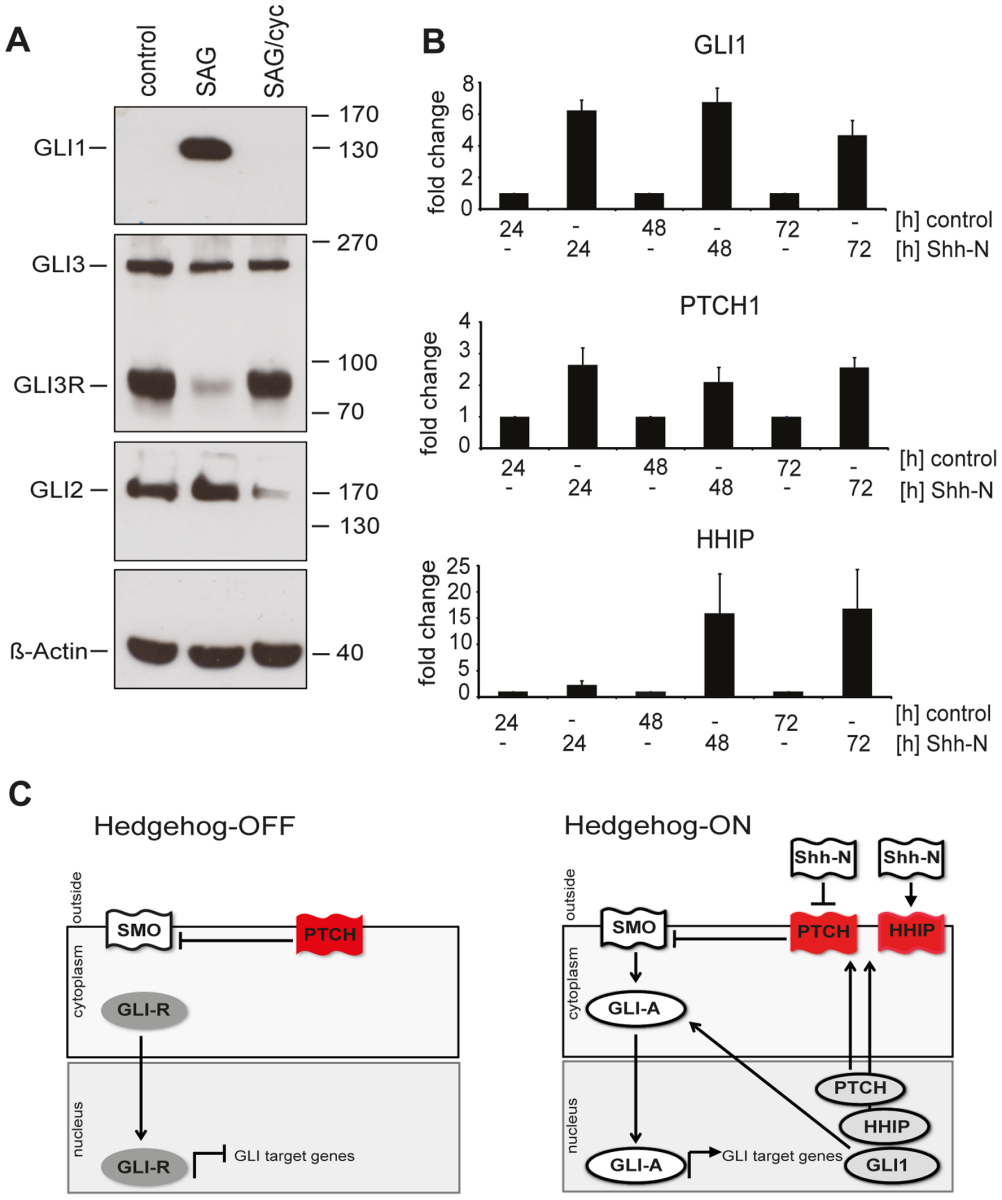
Daoy cells respond to SAG and Shh-N by upregulating canonical HH/GLI targets. (A) Incubation with SAG (100 nM) induced the expression of GLI1 protein and inhibited processing of endogenous GLI3 to its repressor form. Co-incubation with Cyclopamine (cyc, 5 µM) inhibited the SAG-induced GLI1 expression and promoted processing of GLI3 to its repressor (GLI3R) form and inhibited GLI2 expression. Beta-actin shown as Western blot loading control. (B) Transcripts for GLI1 and PTCH were upregulated after stimulation with Shh-N for 24 h, induction of HHIP transcripts was seen after 48 h. Enhanced GLI1, HHIP, and PTCH expression levels were still observed after 72 hours. (C) Schematic presentation of canonical Hedgehog signaling. Left panel illustrates silencing of Hedgehog-mediated signaling via a PTCH-mediated block of SMO so that repressor GLI3 prevails and limits the expression rate of Hedgehog target genes. The right panel illustrates the “Hedgehog-on” state, activating GLI proteins now control the expression of Hedgehog-target genes such as GLI1, PTCH, and HHIP. Upregulation of PTCH and HHIP will eventually result in the downregulation of Hedgehog-signaling. Red color indicates a protein with inactivating properties, white color indicates a protein with activating properties, and grey color indicates RNA transcripts.

The Daoy human medulloblastoma cell line displayed a fully responsive HH pathway. Responsiveness to activation of Shh-signaling was concluded with the following observations (i) accumulation of GLI1 protein and concurrent inhibition of GLI3 repressor formation after stimulation with SAG (Figure 1 A), (ii) processing of GLI3 to its repressor form GLI3R and abrogation of GLI1 and GLI2 protein expression after inhibition of SAG-stimulated Daoy cells with cyclopamine (Figure 1 A), (iii) induction of HH target gene expression (GLI1, HHIP, PTCH) in response to Shh-N treatment (Figure 1 B). Thus, data obtained on the transcriptomic and proteomic level document canonical HH/GLI signaling in response to Shh-N ligand or SMO agonist stimulation in Daoy cells. Therefore, Daoy cells present a valuable human cellular cancer model system for detailed analysis of physiological HH-signaling (Figure 1 C).

**Figure 2:**
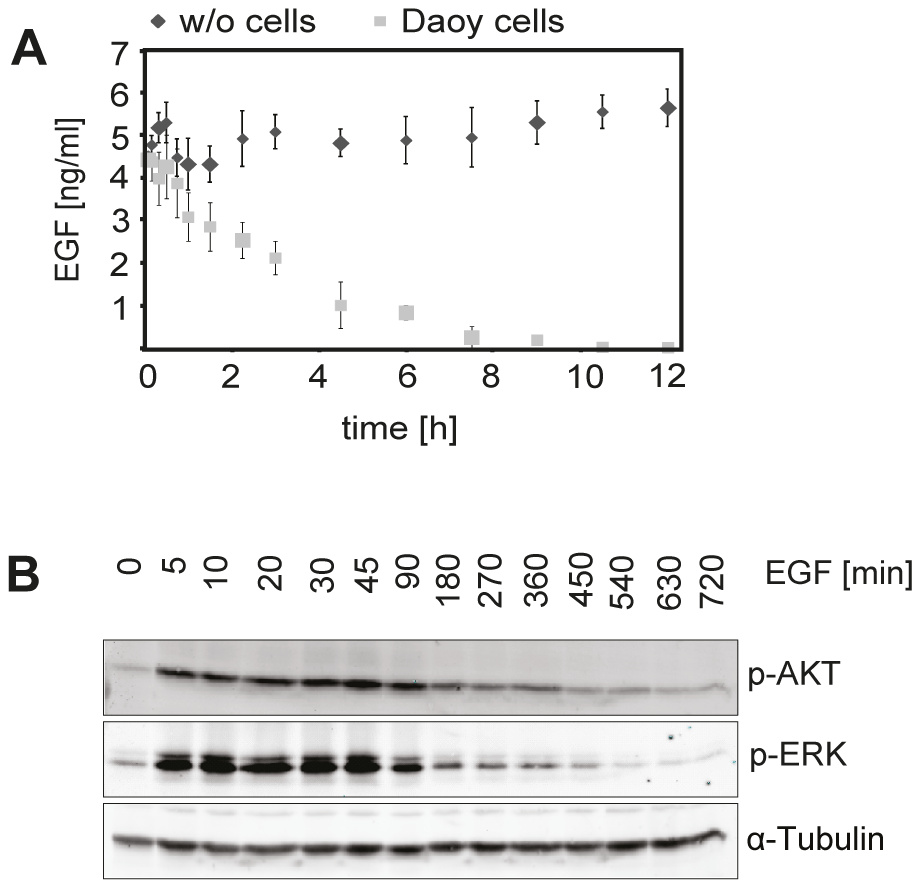
Uptake of EGF by Daoy cells and time-resolved analysis of EGF- downstream signaling. (A) To measure the EGF receptor binding and depletion of the growth factor from the medium supernatant a fluorescent ELISA was performed. Daoy cells were treated with 5 ng/ml of recombinant EGF, and medium supernatants were collected after the indicated time periods (light grey symbols). To control that the ligand is bound and taken up by the cells, and not just degraded by the incubation conditions within the cell culture incubator, wells without cells served as controls (dark grey symbols). (B) Activation of EGF downstream pathways (PI3K/AKT and MAPK) was analyzed by Western blot with phospho-specific antibodies against AKT and ERK. Prior to the application of 5 ng/ml EGF ligand the cells had been starved in low serum (0.5% FBS) medium o/n to minimize basal pathway activation. After the addition of EGF cell lysates were prepared at the indicated timepoints. One sample remained untreated and served as control. Loading of equal protein amounts was checked by probing the Western blot membranes with ß-Tubulin.

Since this study aimed to analyze synergistic events between EGFR and HH-mediated signaling, experimental parameters were optimized to stimulate the EGFR pathway under culture conditions that are required to induce HH signaling in Daoy cells, e.g., cells growing as a highly confluent cell layer (figure S2). EGF ligand uptake by Daoy cells was rapid (Figure 2 A), and correlated with a transient phosphorylation of effector proteins (Figure 2 B). For experiments addressing the crosstalk between both pathways a concentration corresponding to 5 ng/ml EGF was chosen to avoid off-target or saturation effects (figure S2).

### Canonical Hedgehog Target Genes are Downregulated after Activating EGFR Signaling in Shh-N Primed Cells in a MEK1/2- and PI3K-independent Manner

To further elucidate the crosstalk between EGFR and Shh-N signaling, time-resolved expression profiling experiments were carried out using four different treatment conditions: EGF alone, Shh-N alone, a combination of both ligands, as well as a control without ligands. Samples were collected at multiple time points over a treatment period covering 24 h after EGF addition. Stimulation experiments with single ligands served as references to identify effects resulting from cooperation between both pathways.

RNA obtained at 14 different time points was analyzed by whole genome expression profiling to identify differential effects between combinatorial Shh-N/EGF treatment and single stimulations**.** Pair-wise comparisons between stimulation control and EGF or Shh-N treatment, and co-treatment with both ligands were carried out, yielding a list of 3,827 cooperation response genes (CRG) synergistically up- or downregulated in response to co-treatment. Selected CRGs with a documented role in EGFR or HH signaling were chosen for further validation by qPCR, ELISA or Western blotting [25] (Table 1).

**Table 1:** Targets differentially regulated in response to Shh-N/EGF-crosstalk.

gene symbol	net-effectIllumina	net-effectqPCR	net-effectprotein
IL8	up	up	up
VEGFA	up	up	up
MMP7	up	up	up
MMP9	up	up	n.d.
MMP2	up	up	n.d.
IL7R	up	up	n.d.
SOX2	down	down	n.d.
BCL2	n.a.	down	n.d.
GLI1	down	down	unchanged
GLI3	down	down	up
PTCH1	down	down	n.d.
HHIP	n.a.	down	n.d.

n.a.: no corresponding Illumina probe.

n.d.: protein expression not assessed by Western or ELISA.

**Figure 3:**
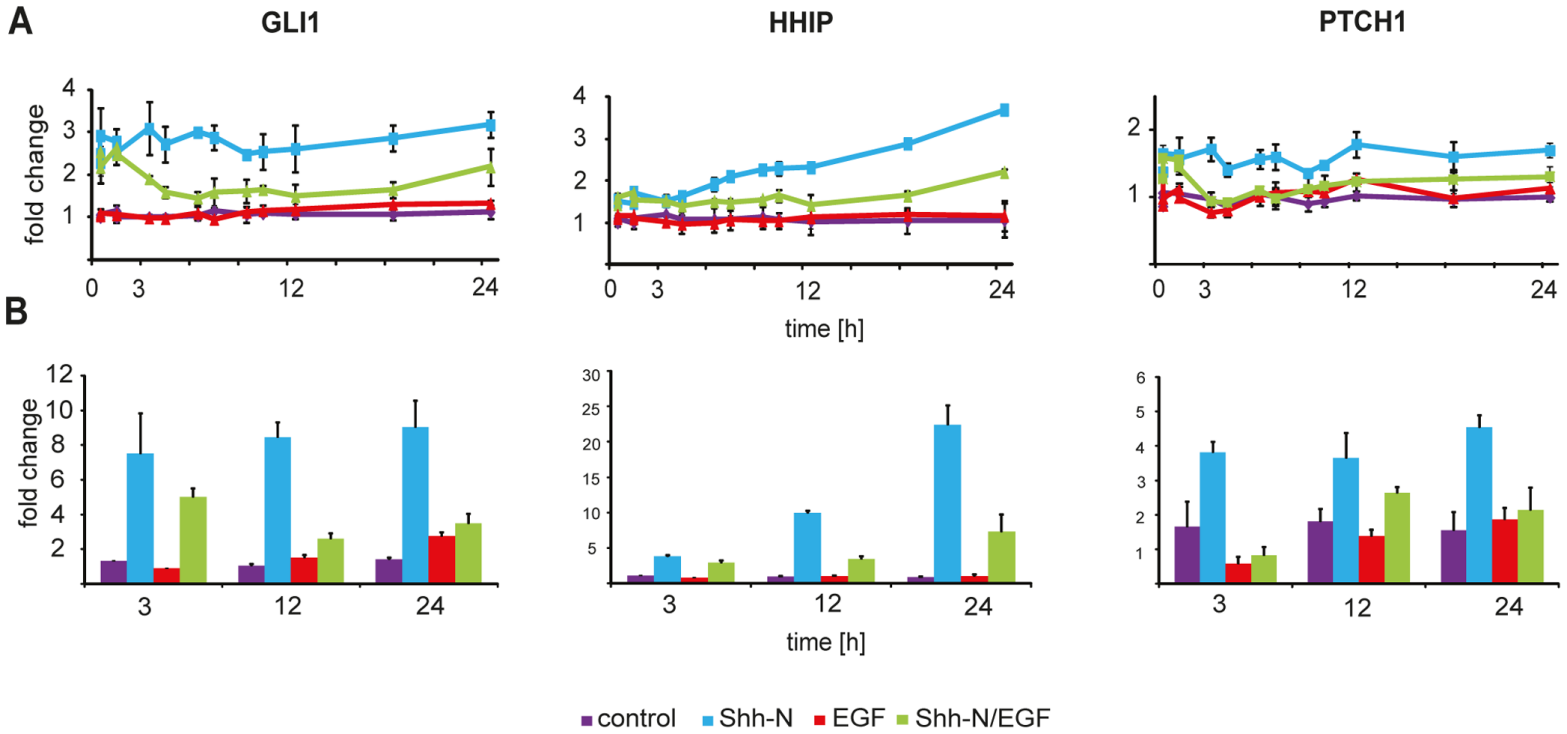
Stimulation of Hedgehog-primed Daoy cells with EGF induced downregulation of canonical HH/GLI targets. Daoy cells were either treated with control medium (purple), Shh-N conditioned medium (blue), EGF (red) or a combinatorial treatment with Shh-N medium and EGF (green) treated for 24 h. (A) Transcript dynamics for GLI1, PTCH, and HHIP were analyzed by transcript profiling on whole genome arrays. Curves represent mean values of independent biological experiments (n = 3, +/−SEM). (B) Taqman RT-PCR confirmed downregulation of canonical Hedghog targets by EGF.

Interestingly, we observed that co-stimulation with Shh-N and EGF resulted in the downregulation of canonical HH/GLI targets (Figure 3). More specifically, repression of GLI1 and PTCH transcript levels was detected within three hours after EGF stimulation of Shh-N pre-exposed cells. Repression of HHIP transcript levels was visible at later time points, which was in line with the fact that HHIP induction occurred 3–6 hours after induction of GLI1 and PTCH. In summary, stimulation with EGF limited the transcriptional upregulation of GLI1 and of genes known to function as negative regulators of HH-signaling, HHIP and PTCH (Figure 3, figure S4).

**Figure 4:**
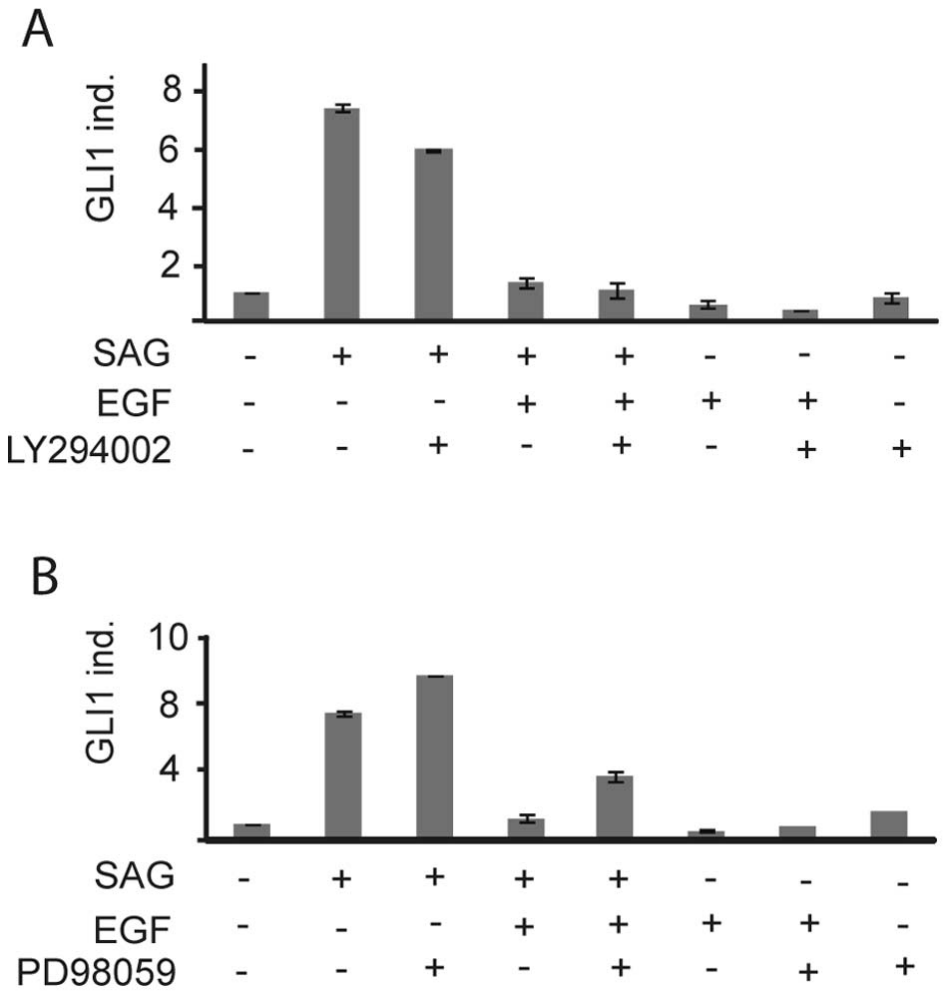
Downregulation of GLI1 as canonical Hedgehog-induced transcript was not rescued after inhibition of MEK/ERK and PI3K/AKT signaling. (A) Impact of PI3K inhibition using LY294002 on the EGF-induced downregulation of GLI1 transcripts after stimulation of Shh-N primed cells for 3 h with EGFR ligands. (B) Impact of MEK1 inhibition using PD98059 on the EGF-induced downregulation of GLI1 transcripts measured 3 h after ligand stimulation of Shh-N primed cells. Figure S3 shows data from a corresponding experiment using U0126 an inhibitor of MEK1/2 and PI103 as a PI3K inhibitor.

As shown in Figure 2 B, induction of EGFR-mediated signaling strongly activated ERK1/2 and AKT. Both kinases are major downstream targets of EGFR signaling, and were therefore considered as likely candidates contributing to the downregulation of GLI1 on the transcript level. Priming of Daoy cells with SAG induced an approximately eight-fold upregulation of GLI1 on the transcript level, which was reduced to two-fold after stimulation with EGF. MEK/ERK and PI3K/AKT signaling, downstream of EGFR, was inhibited using PD98059 and LY294002 as specific inhibitors of MEK1 and of PI3K, respectively. However, neither one of the two inhibitor compounds could rescue GLI1 transcript repression (Figure 4). Comparable results were obtained when MEK/ERK signaling was inhibited with the pan-MEK inhibitor U0126, and PI3K with PI103 (figure S3).

### Synergistic Upregulation of Canonical EGF Target Genes Contrasts Downregulation of Canonical HH Target Genes

**Figure 5:**
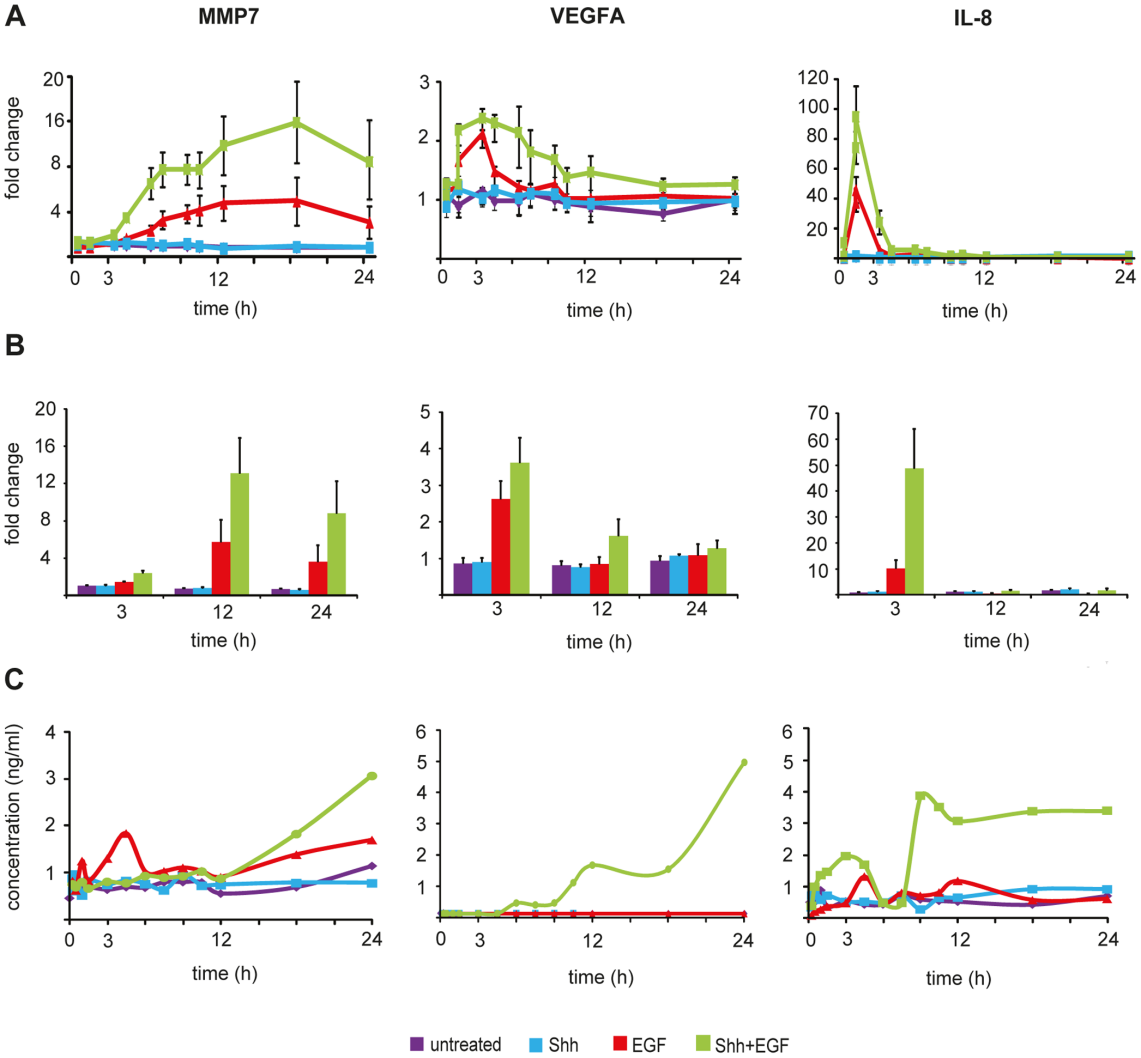
EGF stimulation of Hedgehog-primed Daoy cells strongly upregulated canonical EGF target genes. Cells were treated with either control medium (purple), Shh-N conditioned medium (blue), EGF (red) or a combinatorial treatment with Shh-N medium and EGF (green). (A) Gene expression profiling data indicated upregulation of MMP7, VEGFA, and IL-8. (B) Taqman Real Time PCR confirmed upregulation of canonical EGF targets in Hedgehog-primed cells. (C) MMP7, VEGFA, and IL-8 protein levels were quantified by ELISA in cell culture supernatants at indicated time points. Average fold-changes or concentrations were obtained as mean of three independent biological experiments (+/−SEM).

To further elucidate crosstalk mechanisms between EGF and Shh-N signaling, changes in expression levels were also monitored for known EGF target genes. Contrasting the downregulation of canonical HH targets, and in line with our previous data on human epidermal cells, well-known EGF target genes were observed as synergistically upregulated in response to Shh-N/EGF pathway co-stimulation. Expression profiling data (Figure 5 A) and validation by qPCR (Figure 5 B) revealed that among the top upregulated genes were target genes with well-documented tumor-promoting activities such as matrix metalloproteinases, e.g. MMP7 and MMP9, chemokine receptors such as IL-7R, and proangiogenic and proinflammatory factors such as VEGFA, and IL-8 (Table 1, Figure 5, figure S3). Upregulation of MMP7, VEGFA, and IL-8 was also confirmed at the protein level by ELISA (Figure 5 C). MMP7 protein levels increased 9–12 hours after Shh-N/EGF stimulation, and reached two-fold higher levels under co-treatment conditions, compared to stimulation with EGF alone (Figure 5 C). Although IL-8 and VEGFA transcripts were simultaneously upregulated 1.5 h after EGF stimulation, release of both proteins into the cell culture supernatants followed very different kinetics: IL-8 release occurred 3–6 hours after co-stimulation, whereas extracellular VEGFA levels started to rise hours later reaching a maximum after 18–24 hours of co-stimulation (Figure 5 C). IL-8 expression remained high over the full time period of 24 hours. To functionally validate the release of VEGFA into the cell culture supernatant, a tube formation assay was carried out, using media supernatant obtained 24 hours after co-stimulation (figure S5). Data obtained using a serial dilution of VEGFA suggested that formation of angiogenic vessels is clearly controlled by the VEGFA dosage over a sixteen-fold concentration range. IL-8 could not be confirmed as a pro-angiogenic cytokine in our assay, as confirmed by conflicting published data regarding the role of IL-8 in angiogenesis [29] [30] (figure S5). VEGFA and IL-8 concentrations in media supernatants collected 24 hours after co-stimulation corresponded to 1.7 ng/ml and 1,000 ng/ml, respectively, as determined by ELISA. However, no clear result was obtained when media supernatant was tested in our tube formation assay, possibly due to the low VEGFA concentration (data not shown).

### Synergy between EGFR and Hedgehog-signaling Stabilizes GLI1 on the Protein Level in a MEK1/2 and PI3K Independent Manner

To investigate whether ERK1/2 and PI3K signaling directly influence GLI protein stability and processing, Western blot experiments were carried out. As long as the HH-pathway was silent, repressor GLI3 protein was observed as the dominant GLI3 form (Figure 1 A). In Shh-N primed Daoy cells, the GLI3 repressor form disappeared so that GLI3A/GLI3R ratios increased and GLI3A became the dominant GLI3 isoform (Figure 1 A). A quantitative and is well-accepted method to directly assess GLI activation in response to pathway activation is determining the ratio between full-length activator GLI3 (GLI3A) and repressor GLI3 (GLI3R) [31] [32] [33]. Processing of GLI3A to GLI3R did not change after initiation of EGFR signaling in naïve unprimed Daoy cells as shown over a period of 18 hours (figure S6 A). To support the finding that EGFR signaling stabilizes GLI1 expression without affecting GLI3A/GLI3R ratios, the impact of EGFR signaling on GLI1 protein stability was assessed over an extended time period of 32 hours, and a continuous SAG-induced upregulation of GLI1 unaffected by EGF costimulation was observed (figure S6 B–D).

**Figure 6:**
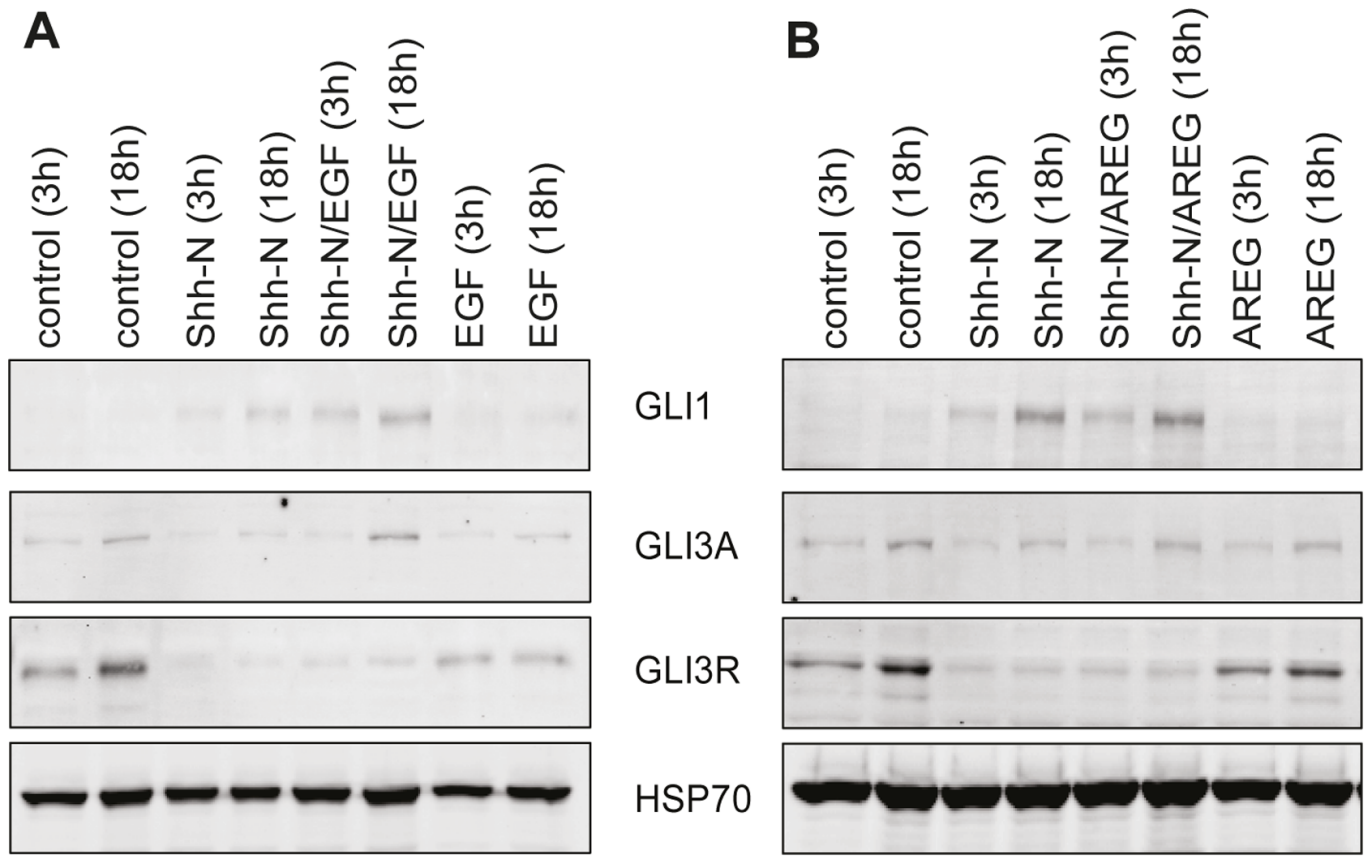
Hedgehog/EGF crosstalk results in maintenance of high GLI1 levels without affecting GLI3 processing. Samples *were* obtained after inducing EGFR signaling in Shh-N primed Daoy cells (Shh-N) or unprimed Daoy cells (EGF) for 3 h or 18 h with EGF (A) or AREG (B). Control cells were neither primed with SAG nor exposed to EGF. Western blots were probed with antibodies against GLI1, GLI3 and HSP70. Figure S6 A shows GLI3A/GLI3R ratios for crosstalk induced by EGF and AREG.

To further strengthen our findings, EGF signaling was induced using Amphiregulin (AREG) instead of EGF. AREG and EGF present two of seven different EGFR ligands [34]. Ligand-binding results in receptor phosphorylation, and initiation of downstream signaling cascades regulating numerous cellular processes such as proliferation, migration, differentiation, and survival, and dysregulated EGFR signaling plays a role in tumorigenesis. AREG has been reported to be overexpressed in aggressive forms of cancer, and distinct signaling properties have been reported for EGFR and AREG with respect to endocytosis of ligand-receptor complexes, and initiation of downstream signaling. For example, AREG induces phosphorylation of EGFR on Y1045 to a lesser extent than EGF extending the cellular half-life of the receptor [35]. However, despite numerous differences, AREG mimicked the effect of EGF on canonical Hedgehog-target genes, and confirmed that EGFR signaling indeed results in a stabilized expression of GLI1, even over longer periods of time (figure S6B–D) and after inhibiting MEK/ERK or PI3K/AKT signaling (figure S7). Additionally, no differences between EGF and AREG were observed for GLI3A/GLI3R formation (figure S6 A). Hence, the induction of EGFR signaling by AREG or EGF in HH- or SAG stimulated Daoy cells allows stable production of GLI1 protein despite a downregulation of GLI1 transcript levels. In line with these findings, EGFR signaling did not impact GLI3A/GLI3R ratios in the HH-on state (Figure 6).

### No Direct Interactions Exist between EGF and HH-mediated Events on the Signaling Level, Indicating that Cooperative Effects between both Pathways are Mediated on the Transcriptional Level

**Figure 7:**
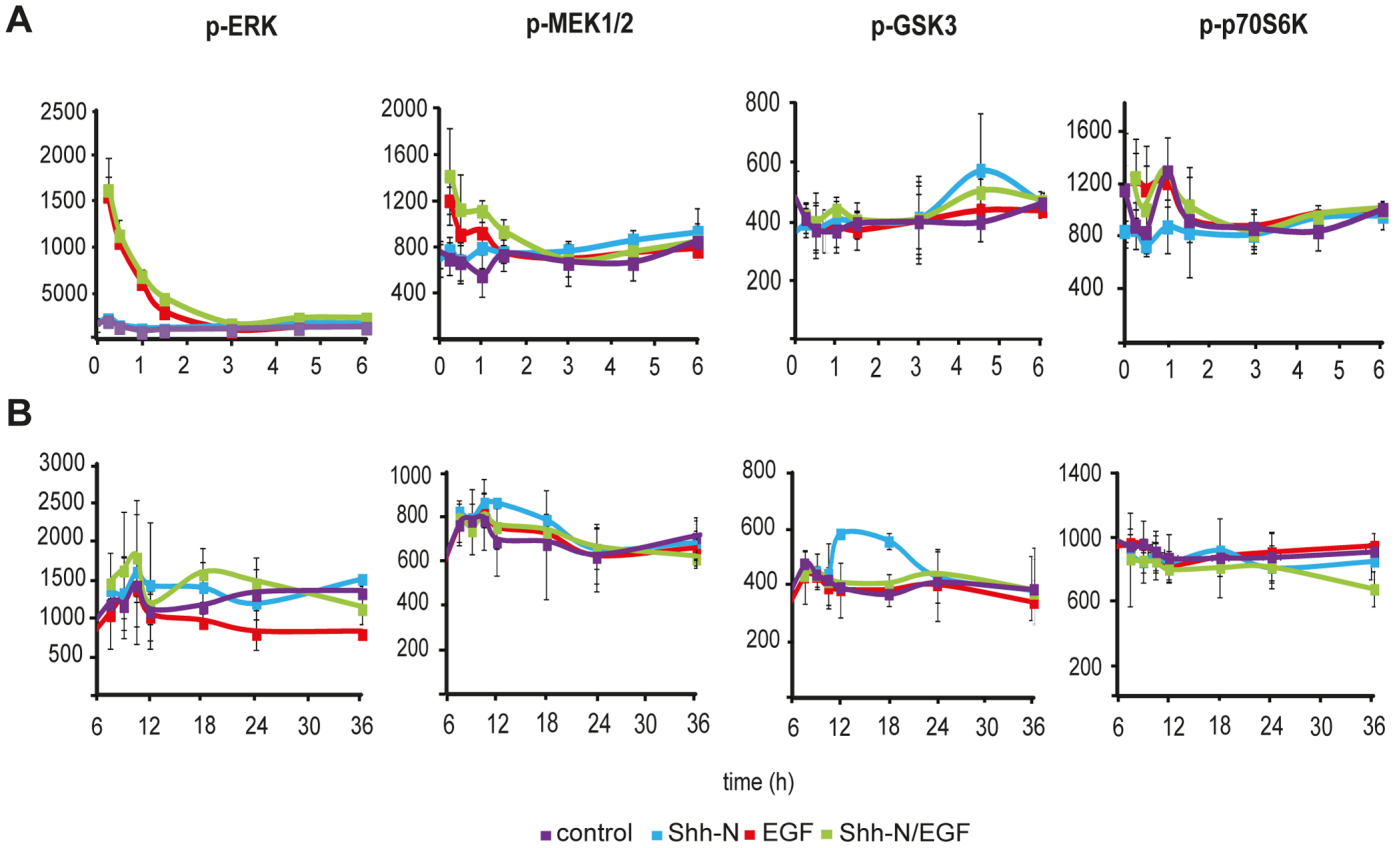
Hedgehog/EGF crosstalk analyzed on the signaling level by RPPA. Samples *were* obtained at 14 time points after inducing EGFR signaling in Shh-N primed Daoy cells or control Daoy cells by EGF. Daoy cells were either treated with control medium (purple), Shh-N conditioned medium (blue), EGF (red) or a combinatorial treatment with Shh-N medium and EGF (green) treated for 24 h. Data for selected phosphoproteins are shown for short time signaling (A: 0–6 h) and long term signaling (B: 6–24 h). Certain kinases, e.g. MEK and ERK1/2, clearly depend on fast signals mediated by EGFR, most proteins assessed by RPPA show no significant changes on the phosphoprotein level as shown here for p70S6K and GSK3 signaling. Table 2 summarizes phosphoproteins probed by RPPA.

**Figure 8:**
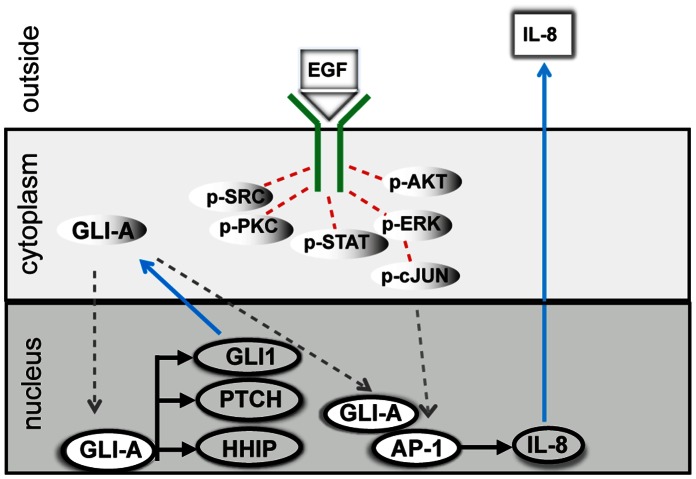
. **Molecular interactions between Hedgehog and EGF signaling pathways occur on the transcriptional level.** Rectangular symbols indicate proteins, oval symbols reflect transcripts. Protein translocation is shown as dashed grey lines, ligand-induced activation of signaling pathways mediated by phosphorylation events is shown as dashed red lines and induction of protein expression as blue lines. Proteins and/or transcripts are produced in response to EGF and/or Shh-N signaling. Release of IL8 is shown as an example of crosstalk induced enhanced secretion of tumor-promoting inflammatory extracellular proteins. GLI1-A and AP-1 interact on the transcriptional level to enhance production of canonical EGF target genes. Numerous phosphoproteins are activated by EGFR-signaling with PI3K/AKT and MEK/ERK signaling as major downstream signaling events. However, both pathways do neither contribute to a stabilization of GLI1 on the protein level nor do they contribute to the observed silencing of canonical Hedgehog target genes on the transcript level. Low activity EGFR signaling events such might play a role in this process or might be mediated by EGFR-induced miRs.

To assess whether synergistic effects result from direct cooperation between HH/EGF-mediated responses, time-resolved measurements were extended and protein lysates were obtained matching time points described for transcript profiling. Targeted proteome profiling was carried out using reverse phase protein arrays (RPPA), in order to assess the activation state of major signaling pathways downstream of EGFR (Table 2). Among >30 proteins probed by RPPA, we found no indication that would point towards synergistic effects between both pathways on the signaling level. Data was analyzed for 6 hours (Figure 7 A), showing fast signaling events, and after longer time periods of 24 hours (Figure 7 B). The regulation of phosphoproteins in response to HH-EGFR activation is summarized in Table 2. RPPA data showed that the activation of MEK/ERK signaling was clearly EGF-dependent. Since HH-signaling did not show any direct impact on signaling pathways downstream of EGFR, the data implies that synergistic events are integrated at the level of transcriptional control, similar to mechanisms of HH-EGFR integration in epidermal cells [6] (Figure 8).

**Table 2:** Phosphoproteins quantified by RPPA.

	Control	EGF	Shh-N	Shh-N/EGF
pMEK1/2	0	up	0	up
pERK1/2	0	up	0	up
pAKT (S473)	0	up	0	up
pp70S6K	0	0	0	0
pGSK3ß	0	0	0	0
pRSK1–3	0	0	0	0
pPLCγ (Y771)	0	0	0	0
pPLCγ (Y783)	0	0	0	0
pcJUN (S63)	0	up	0	up
pcJUN (S73)	0	up	0	up
E-Cadherin	0	up	0	up
pmTOR (S2448)	0	0	0	0
pRPS6 (S235/236)	0	up	0	up
pCREB (S133)	0	0	0	0
pRB (S807/811)	0	0	0	0
pNFκB (S276)	0	0	0	0

## Discussion

Development and progression of malignant transformation is a multistep process, caused by molecular aberrations affecting different cellular signaling pathways [36]. This also applies to the HH/GLI signaling pathway, however, tremendous efforts are required to unravel regulatory mechanisms occurring from interactions among different signaling modules [37] [8]. The aim of this study was to examine HH-mediated signaling under physiological conditions in a human model system, and to assess interactions between HH and EGF-mediated signaling. Initially, we needed to identify a human cell line with a fully responsive HH pathway that could serve as model system of Hedgehog signaling. Employing a qPCR screen, we identified a high-level expression of the HH-regulated receptor SMO in Daoy cells, and chose to establish a protocol for direct stimulation with SMO agonist (SAG), and of PTCH, a negative regulator of SMO, with Shh-N conditioned medium.

Daoy cells were employed as the model system to identify tumor-relevant cooperation response genes (CRGs), which were synergistically regulated by combined HH and EGFR signaling. Two different modes of molecular regulation initiated by the HH/EGF cross talk were identified, with relevance for tumor initiation and tumor progression. More specifically, tumor-promoting target genes, e.g. MMP7/9, IL-8 and VEGFA, were synergistically upregulated, while intriguingly negative modulators of HH/GLI signaling, such as HHIP and PTCH1, were downregulated. Repression of the negative pathway regulators was accompanied by a continued high-level expression of GLI1 protein, despite its fast downregulation on the transcript level. Our findings on GLI1 protein expression are in line with reports of the positive impact of receptor tyrosine kinase-induced signaling on the nuclear localization, transcriptional activity, and protein stability of this protein [4] [11] [13]. In contrast, Fogarty et al. reported that bFGF-mediated signaling reduces GLI transcriptional activity, and concluded that this was caused by the bFGF-induced activation of MAPK and JNK signaling [20]. Thus, the bFGF example suggests that activation of receptor tyrosine kinase-driven pathways can also have a dampening effect on GLI-mediated transcription. However, Daoy cells do not respond to FGF (data not shown), so that a direct comparison between Daoy cells and data presented by Forgarty and co-authors could not be carried out to clarify this point. These findings also underline the diversity of receptor-tyrosine kinase-induced signaling, and indicate a differential impact on HH/GLI signal modulation.

Hence, we concluded that long-term GLI1 protein stability, as seen in Daoy cells, might reflect a HH-signaling signaling past. Whether EGFR signaling mediates a direct repression of negative HH pathway regulators PTCH and HHIP presents a likely hypothesis that needs further validation. GLI1, as a transcription factor, can likely contribute to the modulated transcriptional response observed after inducing EGFR signaling. Increased levels of GLI1 protein might remain undetected when merely relying on transcriptomic profiling tools: this is especially true for cell or tumor types with activated EGFR signaling. Taken one step further, our findings also suggest that GLI1 itself presents a promising therapeutic target [38], and targeting GLI1 in certain cases might be superior to targeting SMO. Our findings also point towards the fact that a stabilized expression of GLI1 as independent of PI3K/AKT- and MEK/ERK signaling, and might hence be induced by other EGFR-signaling modules. For example, Lauth et al. [18] reported that impact of PKCδ on the Hedgehog pathway stabilizes GLI1 on the protein level, which was demonstrated as independent of MEK1 in line with our findings. Furthermore, Jones and co-authors also recently suggested a yet unrecognized role for low-affinity receptor signaling, mediated by SRC or by PLC-γ, might play a significant, yet unexpected, role in the propagation of ERBB signals [39].

Synergism between HH/GLI and EGFR signaling also accounted for a release of proteins, such as IL-8, as an early event, and of MMP7 and VEGFA at later time points. These three proteins have a well-documented role in driving tumor progression and metastasis. Besides a function of MMP proteins in matrix degradation [40], MMP7 has been implicated in the release of bioactive molecules, such as IGF, by playing a major role in medulloblastoma pathogenesis [41] [42]. MMP7 is also involved in the regulation of angiogenesis in its releasing of VEGF [43] [44]. This finding is in line with an increased production, and sustained release of VEGFA in response to HH-EGFR crosstalk, and can possibly be ascribed to a functional interaction between MMP7 and VEGFA. In general, it has been shown that malignant brain tumors are highly angiogenic, as compared to other types of solid human tumors, and produce a variety of proangiogenic factors [45] [46]. A prominent and fast upregulation of IL-8 was observed, although neo-angiogenic properties of IL-8 could not be confirmed in our tube formation assay. Nevertheless, in the tumor microenvironment IL-8 is a key regulator for the recruitment of infiltrating neutrophils, which further promotes tumor metastasis [47]. As the aforementioned tumor-promoting factors identified in this study are secreted proteins, it will be important to determine their auto- or paracrine-signaling activity, and to address crosstalk with stroma cells under physiological conditions in future studies. In summary, our results describe novel aspects of HH-EGFR signal cooperation, and provide evidence for a molecular model in which MMP7, IL-8, and VEGFA are induced and secreted at high levels in response to cooperative interactions between HH/GLI and EGFR signaling. According to the well-documented role of MMP-7, IL-8, and VEGFA for cancer development and tumor growth, synergistic effects between HH/EGF mediated signaling pathways may accelerate tumor initiation and support tumor growth.

## Supporting Information

Figure S1 **Characterization and validation of Shh-N conditioned medium.** (A) Western blot analysis confirmed the presence of Shh-N in cell culture medium obtained after transfecting HEK293FT with a Shh-N expression plasmid. Standard growth medium (DMEM +10%FBS) and medium supernatant from cells transfected with an empty vector served as negative controls. (B) Luciferase reporter assays were carried out in SHH-Light II cells to test the biological activity of Shh-N conditioned medium. Cells were treated for 48 h with standard growth medium (DMEM +10%FBS), control medium, 20% or 40% of Shh-N conditioned medium, a combination of Shh-N medium and different amounts of Shh-N 5E1 blocking antibody or different amounts of 5E1 alone.
(EPS)

Figure S2 **Response of Daoy cells to stimulation with EGF.** (A) Indicated concentrations of EGF were applied for 15 min to cells starved o/n. Activation of EGFR and downstream pathways (PI3K/AKT and MAPK) was analyzed with phospho-specific antibodies against EGFR, AKT and ERK1/2. 2.5 ng/ml EGF was chosen for further experiments. (B) The influence of cell density on EGF-induced signaling was assessed. Daoy cells were seeded on the same day and kept in culture for several days as indicated. Cells were starved with low serum (0.5% FBS) medium o/n. Stimulation of EGF driven pathways was induced by application of 2.5 ng/ml for 15 min. After stimulation, cells were harvested and equal amounts of cell lysates were used for Western blot analysis. Activation of EGFR and downstream pathways (PI3K/AKT and MAPK) was analyzed with phospho-specific antibodies against EGFR, AKT and ERK 1/2. Loading of equal amounts of protein was controlled with anti-actin antibody.
(PDF)

Figure S3 **SAG/EGF-induced downregulation of GLI1 was not rescued after inhibiting EGFR-mediated signaling using inhibition of MEK/ERK and PI3K/AKT.** PI3K/AKT signaling was inhibited using PI103 and MEK1/2 signaling was inhibited using U0126, mRNA was obtained 3 h after stimulation of SAG primed cells with EGF.
(PDF)

Figure S4 **SOX2 and BCL2 as Hedgehog-driven genes are downregulated by EGF co-stimulation while the expression of canonical EGF target genes such as MMP9 and IL7R is amplified.** (A–D) Daoy cells were either treated with control medium (purple), Shh-N conditioned medium (blue), EGF (red) or Shh-N medium plus EGF (green) to activate the respective signaling pathways. Total RNA was prepared at the indicated timepoints. (A) and (C) HumanHT-12 v4 chips were used for expression profiling. Signal readout was achieved by scanning of chips with an appropriate scanner using the BeadScan Software. Normalized signal intensities from each independent biological experiment were used to calculate fold change ratios compared to the control treatment sample (t = 0 h) which served as reference. Curves shown represent the mean of independent experiments (n = 3, +/− SEM). (B) and (D) After cDNA synthesis 25 ng of cDNA were applied for Taqman Real Time PCR in combination with the UPL-Probe® system. The estimated expression values of the analyzed target genes in each sample were normalized with the respective amount of the housekeeper gene HPRT. Finally, the normalized expression values of each timepoint/treatment were used to calculate fold change ratios compared to the control treatment sample (t = 0 h) which served as reference. Fold change values shown represent the average expression from independent biological experiments (n = 3, +/− SEM).
(PDF)

Figure S5 **Tube formation assay.** Normal Human Dermal Fibroblasts (NHDF) were seeded in 24-well plates and cultured for 5 days in DMEM supplemented with 10% fetal calf serum, 1% HEPES and 1% non-essential amino acids. Human umbilical cord vein endothelial cells (HUVEC) were seeded on a confluent NHDF layer and different concentrations of VEGFA, VEGFA and/or IL8 were added to the cell culture. The cells were incubated with a monoclonal antibody against the CD31. Microscopic quantitative analysis of tube formation was performed with the software Angiosys 1.0, TCS (Cellworks).
(PDF)

Figure S6 **GLI3A/GLI3R ratios increase in response to Hedgehog-signaling and not regulated by EGFR.** (A) Western blot-based analysis of GLI3A/GLI3R levels. HH/GLI signaling was induced for 24 h, EGFR ligands as indicated were added for 18 h. **GLI1 protein expression was stably produced for at least 32 h.** (B) EGF signaling was induced after 24 h pre-exposure of Daoy cells to SAG. Samples were collected after 3, 6, 18 and 32 h and analyzed by Western blot. (C) AREG signaling was induced after 24 h pre-exposure of Daoy cells to SAG. Samples were collected after 3, 6, 18 and 32 h and analyzed by Western blot. (D) GLI1 signals were normalized to Actin as loading control. No differences were seen for AREG and EGF-mediated signaling on GLI1 stability.
(PDF)

Figure S7 **Inhibition of EGFR-induced PI3K/AKT and MEK/ERK signaling did not influence GLI1 protein stability.** Highly confluent Daoy cells were exposed for 24 h to SAG and incubated for 1 h with LY204002 and PD98059 to inhibit PI3K and MEK1/2, respectively. EGFR signaling was initiated for 18 h by adding AREG or EGF. Cell lysates were collected and analyzed by Western blot. Staining for Actin-ß was used to normalize for equal loading after background correction. No differences were noticed with respect to AREG and EGF-mediated signaling on GLI1 stability.
(PDF)

Table S1 **qRT-Primer information.**
(XLSX)
