# Supplementary figures and images for: Synergism between Hedgehog-GLI and EGFR Signaling in Hedgehog-Responsive Human Medulloblastoma Cells Induces Downregulation of Canonical Hedgehog-Target Genes and Stabilized Expression of GLI1
Source: PLoS One. 2013 Jun 10;8(6):e65403. doi: 10.1371/journal.pone.0065403 (PMC3677915; doi:10.1371/journal.pone.0065403)

# Supplement figure S2

**A**

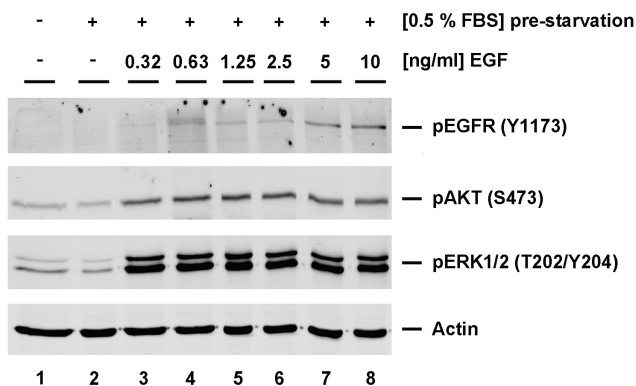

**B**

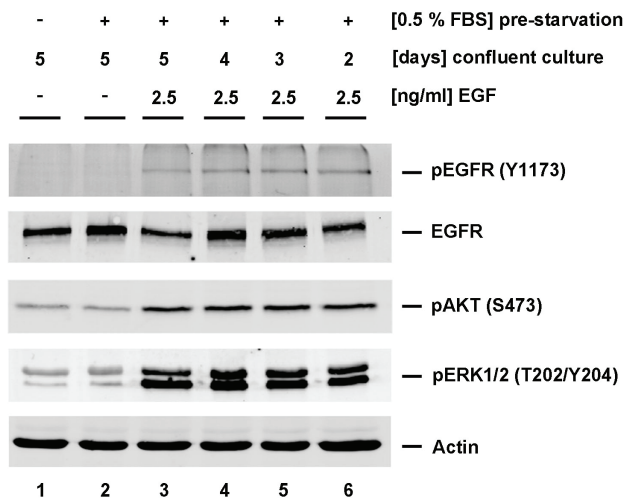

Supplement: Figure S2 — Response of Daoy cells to stimulation with EGF. (A) Indicated concentrations of EGF were applied for 15 min to cells starved o/n. Activation of EGFR and downstream pathways (PI3K/AKT and MAPK) was analyzed with phospho-specific antibodies against EGFR, AKT and ERK1/2. 2.5 ng/ml EGF was chosen for further experiments. (B) The influence of cell density on EGF-induced signaling was assessed. Daoy cells were seeded on the same day and kept in culture for several days as indicated. Cells were starved with low serum (0.5% FBS) medium o/n. Stimulation of EGF driven pathways was induced by application of 2.5 ng/ml for 15 min. After stimulation, cells were harvested and equal amounts of cell lysates were used for Western blot analysis. Activation of EGFR and downstream pathways (PI3K/AKT and MAPK) was analyzed with phospho-specific antibodies against EGFR, AKT and ERK 1/2. Loading of equal amounts of protein was controlled with anti-actin antibody. (PDF) [file pone.0065403.s002.pdf]

## Supplement figure S3

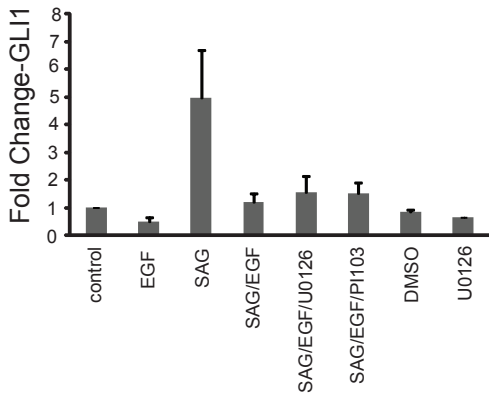

Supplement: Figure S3 — SAG/EGF-induced downregulation of GLI1 was not rescued after inhibiting EGFR-mediated signaling using inhibition of MEK/ERK and PI3K/AKT. PI3K/AKT signaling was inhibited using PI103 and MEK1/2 signaling was inhibited using U0126, mRNA was obtained 3 h after stimulation of SAG primed cells with EGF. (PDF) [file pone.0065403.s003.pdf]

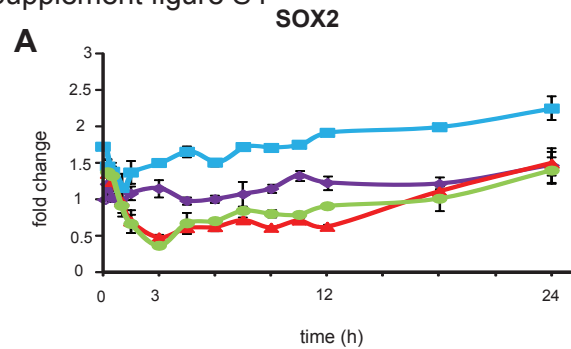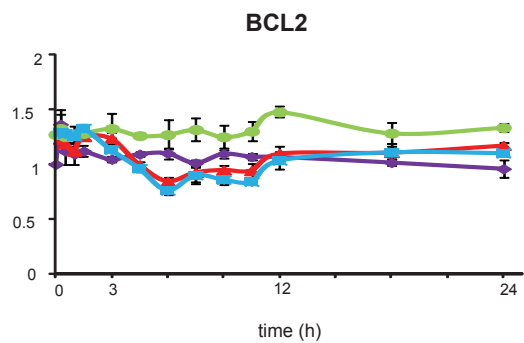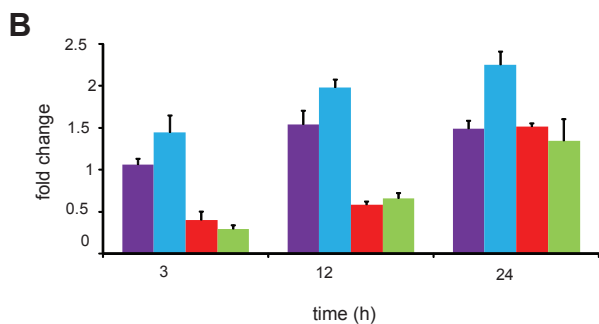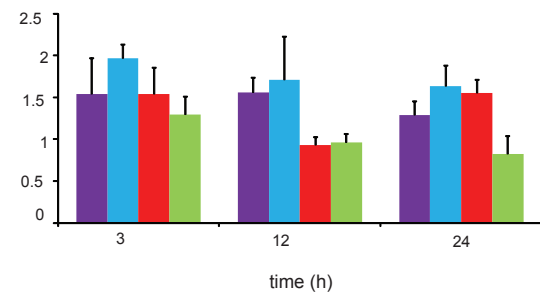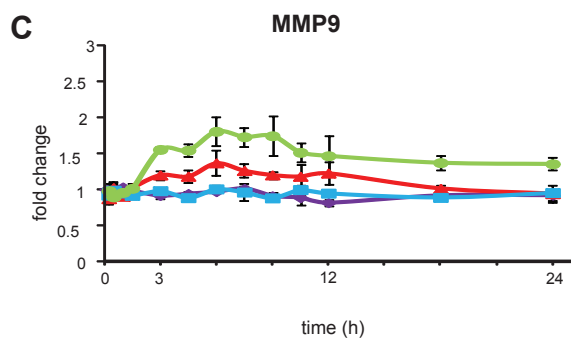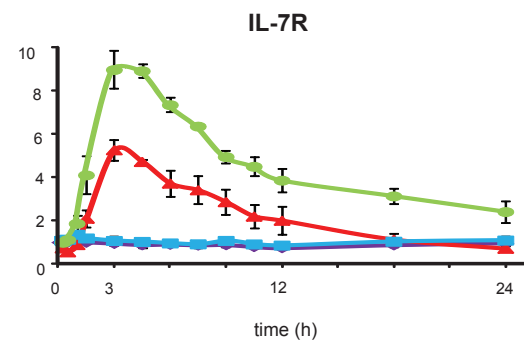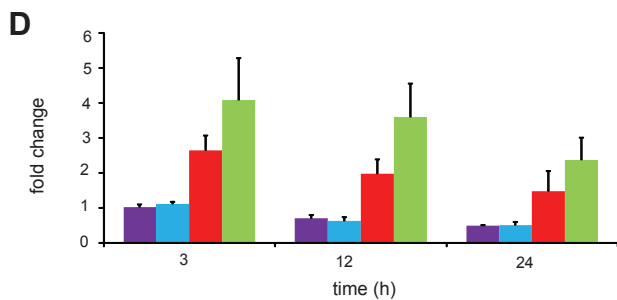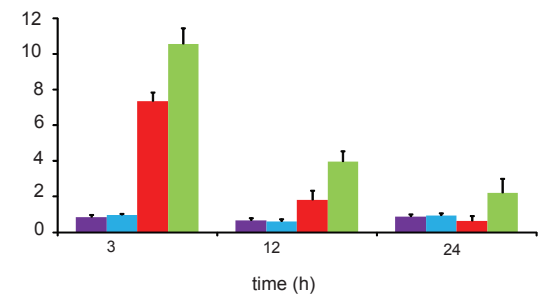

untreated Shh EGF Shh+EGF

Supplement: Figure S4 — SOX2 and BCL2 as Hedgehog-driven genes are downregulated by EGF co-stimulation while the expression of canonical EGF target genes such as MMP9 and IL7R is amplified. (A–D) Daoy cells were either treated with control medium (purple), Shh-N conditioned medium (blue), EGF (red) or Shh-N medium plus EGF (green) to activate the respective signaling pathways. Total RNA was prepared at the indicated timepoints. (A) and (C) HumanHT-12 v4 chips were used for expression profiling. Signal readout was achieved by scanning of chips with an appropriate scanner using the BeadScan Software. Normalized signal intensities from each independent biological experiment were used to calculate fold change ratios compared to the control treatment sample (t = 0 h) which served as reference. Curves shown represent the mean of independent experiments (n = 3, +/− SEM). (B) and (D) After cDNA synthesis 25 ng of cDNA were applied for Taqman Real Time PCR in combination with the UPL-Probe® system. The estimated expression values of the analyzed target genes in each sample were normalized with the respective amount of the housekeeper gene HPRT. Finally, the normalized expression values of each timepoint/treatment were used to calculate fold change ratios compared to the control treatment sample (t = 0 h) which served as reference. Fold change values shown represent the average expression from independent biological experiments (n = 3, +/− SEM). (PDF) [file pone.0065403.s004.pdf]

Supplement figure S5

**A**

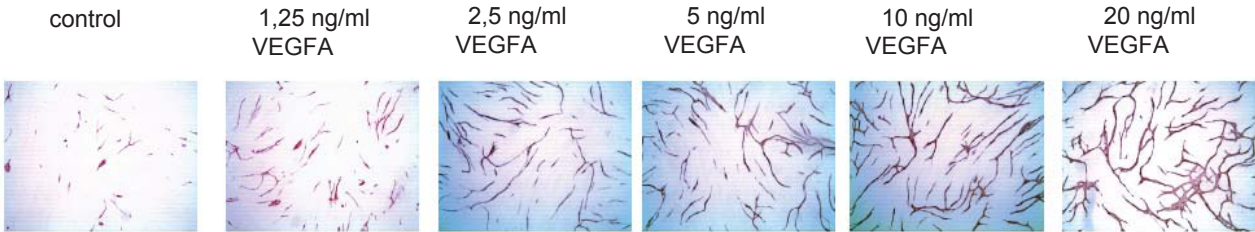

**B**

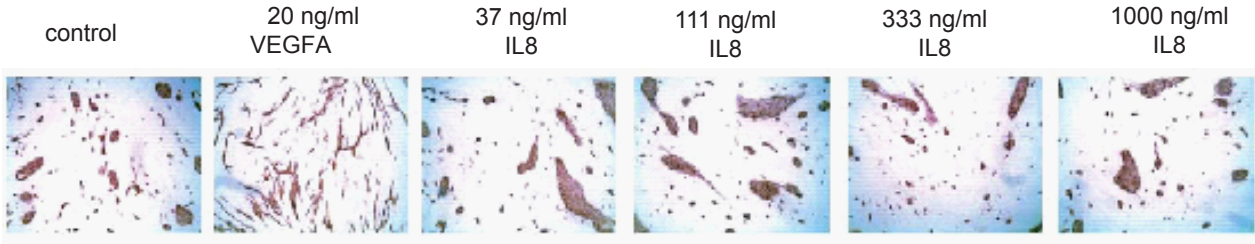

Supplement: Figure S5 — Tube formation assay. Normal Human Dermal Fibroblasts (NHDF) were seeded in 24-well plates and cultured for 5 days in DMEM supplemented with 10% fetal calf serum, 1% HEPES and 1% non-essential amino acids. Human umbilical cord vein endothelial cells (HUVEC) were seeded on a confluent NHDF layer and different concentrations of VEGFA, VEGFA and/or IL8 were added to the cell culture. The cells were incubated with a monoclonal antibody against the CD31. Microscopic quantitative analysis of tube formation was performed with the software Angiosys 1.0, TCS (Cellworks). (PDF) [file pone.0065403.s005.pdf]

# Supplement figure S7

|          |   |   |   |   |   |   |   |   |
|----------|---|---|---|---|---|---|---|---|
| SAG      | - | + | + | + | + | + | + | + |
| Ligand*  | - | - | - | - | - | + | + | + |
| LY204002 | - | - | + | - | + | - | + | - |
| PD98059  | - | - | - | + | + | - | - | + |

AREG\*

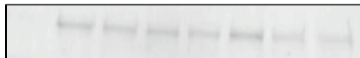

GLI1

EGF\*

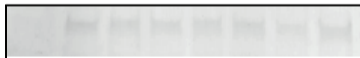

GLI1

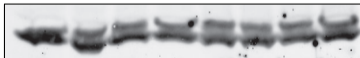

$\beta$ -Actin

Supplement: Figure S7 — Inhibition of EGFR-induced PI3K/AKT and MEK/ERK signaling did not influence GLI1 protein stability. Highly confluent Daoy cells were exposed for 24 h to SAG and incubated for 1 h with LY204002 and PD98059 to inhibit PI3K and MEK1/2, respectively. EGFR signaling was initiated for 18 h by adding AREG or EGF. Cell lysates were collected and analyzed by Western blot. Staining for Actin-ß was used to normalize for equal loading after background correction. No differences were noticed with respect to AREG and EGF-mediated signaling on GLI1 stability. (PDF) [file pone.0065403.s007.pdf]
