# Supplementary material for: Synergism between Hedgehog-GLI and EGFR Signaling in Hedgehog-Responsive Human Medulloblastoma Cells Induces Downregulation of Canonical Hedgehog-Target Genes and Stabilized Expression of GLI1
Source: PLoS One. 2013 Jun 10;8(6):e65403. doi: 10.1371/journal.pone.0065403 (PMC3677915; doi:10.1371/journal.pone.0065403)
Supplement: Figure S6 — GLI3A/GLI3R ratios increase in response to Hedgehog-signaling and not regulated by EGFR. (A) Western blot-based analysis of GLI3A/GLI3R levels. HH/GLI signaling was induced for 24 h, EGFR ligands as indicated were added for 18 h. GLI1 protein expression was stably produced for at least 32 h. (B) EGF signaling was induced after 24 h pre-exposure of Daoy cells to SAG. Samples were collected after 3, 6, 18 and 32 h and analyzed by Western blot. (C) AREG signaling was induced after 24 h pre-exposure of Daoy cells to SAG. Samples were collected after 3, 6, 18 and 32 h and analyzed by Western blot. (D) GLI1 signals were normalized to Actin as loading control. No differences were seen for AREG and EGF-mediated signaling on GLI1 stability. (PDF) [file pone.0065403.s006.pdf]

Supplemental figure S6

A

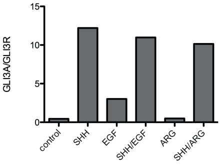

B

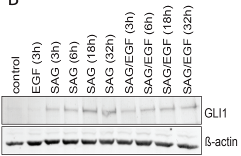

C

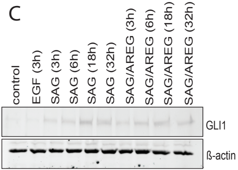

D

Actin-normalized GLI1 signal

	**control**	**SAG only**				**SAG + EGFR ligand**			
[h]	0	3	6	18	32	3	6	18	32
EGF	56	140	158	224	287	158	177	174	281
AREG	92	160	179	308	264	172	239	326	308
